# Gymnosperms on the EDGE

**DOI:** 10.1038/s41598-018-24365-4

**Published:** 2018-04-16

**Authors:** Félix Forest, Justin Moat, Elisabeth Baloch, Neil A. Brummitt, Steve P. Bachman, Steffi Ickert-Bond, Peter M. Hollingsworth, Aaron Liston, Damon P. Little, Sarah Mathews, Hardeep Rai, Catarina Rydin, Dennis W. Stevenson, Philip Thomas, Sven Buerki

**Affiliations:** 1 Royal Botanic Gardens, Kew, Richmond, Surrey, TW9 3AB United Kingdom; 2 School of Geography, University of Nottingham, Nottingham, NG7 2RD United Kingdom; 3 Department of Life Sciences, Natural History Museum, Cromwell Road, London, SW7 5BD United Kingdom; 4 University of Alaska Museum, University of Alaska, 907 Yukon Drive, Fairbanks, AK 99775-6960 USA; 5 Royal Botanic Garden, Edinburgh, Inverleith Row, Edinburgh, EH3 5LR United Kingdom; 6 Department of Botany and Plant Pathology, Oregon State University, 2082 Cordley Hall, Corvallis, OR 97331-2902 USA; 7 The New York Botanical Garden, Bronx, New York, 10458-5126 USA; 8 CSIRO Australian National Herbarium, Centre for Australian National Biodiversity Research, GPO Box 1700, Canberra, ACT 2601 Australia; 9 Centre for Biodiversity Analysis, The Australian National University, Acton, ACT 2601 Australia; 10 Department of Wildland Resources, Utah State University, 5230 Old Main Hill, Logan, Utah 84322 USA; 11 Bergius Foundation, the Royal Swedish Academy of Sciences, and Department of Ecology, Environment and Plant Sciences, Stockholm University, SE-10691 Stockholm, Sweden; 12 Department of Biological Sciences, Boise State University, 1910 University Drive, Boise, Idaho 83725 USA

## Abstract

Driven by limited resources and a sense of urgency, the prioritization of species for conservation has been a persistent concern in conservation science. Gymnosperms (comprising ginkgo, conifers, cycads, and gnetophytes) are one of the most threatened groups of living organisms, with 40% of the species at high risk of extinction, about twice as many as the most recent estimates for all plants (i.e. 21.4%). This high proportion of species facing extinction highlights the urgent action required to secure their future through an objective prioritization approach. The Evolutionary Distinct and Globally Endangered (EDGE) method rapidly ranks species based on their evolutionary distinctiveness and the extinction risks they face. EDGE is applied to gymnosperms using a phylogenetic tree comprising DNA sequence data for 85% of gymnosperm species (923 out of 1090 species), to which the 167 missing species were added, and IUCN Red List assessments available for 92% of species. The effect of different extinction probability transformations and the handling of IUCN data deficient species on the resulting rankings is investigated. Although top entries in our ranking comprise species that were expected to score well (e.g. *Wollemia nobilis*, *Ginkgo biloba*), many were unexpected (e.g. *Araucaria araucana*). These results highlight the necessity of using approaches that integrate evolutionary information in conservation science.

## Introduction

About one in five plants are estimated to be at risk of extinction with a large proportion of these found in tropical rainforests; the main threats are linked to human activities, in particular habitat transformation and harvesting^1,2^. Gymnosperms are among the most threatened living organisms on the planet, with 40% of their species at high risk of extinction, which is about twice as many as the most recent estimates for all plants (i.e. 21.4%)^1^. Which species (and areas) should be prioritised in conservation programmes is a recurrent question, but even more so in groups with such high number of threatened species such as gymnosperms. Phylogenetic diversity (PD)^3^, a metric based on evolutionary history, has been proposed as an approach of choice to establish and implement this prioritization process in conservation. The PD of a group of taxa is equivalent to the sum of all the branches linking the members of this group in a phylogenetic tree, from the root to the tips^3^. Its potential suitability as a representative of feature diversity is one of the most critical characteristics of PD and many have argued that maintaining PD would retain not only the evolutionary potential of species but also the unanticipated benefits for human society (e.g. biodiversity option value^3–8^, but see^9^), One of the various methods emerging from the PD concept that have been proposed to address the prioritization issue in conservation is the Evolutionary Distinct and Globally Endangered (EDGE) approach^10^. It ranks species according to their evolutionary distinctiveness and the level of extinction risk they face. The evolutionary distinctiveness (ED) scores partition the total PD among the species, giving descendent species shared responsibility for internal branches; the total of all ED scores equals the total PD of a tree. It is applied here to gymnosperms after similar lists were generated for mammals^10,11^, birds^12^, corals^13^, amphibians^14^, and sharks and relatives^15^. The only other group of plants for which this approach has been used are cycads^16^. We re-evaluated cycads in the present study in the context of all gymnosperms, augmenting the genetic coverage from 58% to 80% of species. The wealth of genetic data available for gymnosperms, their relatively well-known taxonomy and geographical distributions, and the fact that most species have been evaluated using the IUCN Red List criteria, make this group an ideal candidate for the application of the EDGE method.

Living gymnosperms comprise four distinct lineages, *Ginkgo* (1 spp.), gnetophytes (112 spp.), cycads (339 spp.), and conifers (638 spp.)^17^. The relationships among these groups, as well as their relationships to flowering plants (angiosperms), have been the subject of debates for decades and several, often quite different, hypotheses have been suggested^18^. The existence of numerous lineages known only from the fossil record has contributed to difficulties in deciphering the early evolutionary history of seed plants and different molecular data sets supported incompatible phylogenetic hypotheses^18^. Conifers were furthermore shown to be paraphyletic, with gnetophytes as sister to Pinaceae or to cupressophytes (the non-Pinaceae families Araucariaceae, Cupressaceae, Podocarpaceae, Sciadopityaceae, and Taxaceae)^18–20^. The emergence of molecular tools in phylogenetics failed to bring this debate to a close^18^. Although these uncertainties about the relationships among gymnosperm lineages remain, most recent molecular trees place all living gymnosperms in a monophyletic group sister to angiosperms^20–22^.

Gymnosperms have an extensive fossil record, with relatively well-conserved morphological features exemplified by the numerous species considered living fossils (e.g. *Ginkgo*, *Wollemia*, *Welwitschia*), but species diversity of living gymnosperms is low, with just over 1,000 species compared to the 369,000 found in angiosperms^2^. Families of living conifers and cycads are also younger than the angiosperm crown group^23^ and both the fossil record and molecular phylogenies indicate greater extinction rates in gymnosperms in the Cenozoic^23,24^. Competition with angiosperms may have been a factor^25–28^, but this alone cannot explain the global distribution of gymnosperms^29^. The effective inclusion of the complex evolutionary history of gymnosperms in their conservation planning will be fundamental to the survival of these highly threatened lineages, comprising many peculiar and iconic species of great ecological and economic importance.

## Results and Discussion

We produced a conservation priority list for gymnosperms that accounts for their evolutionary history and extinction risks by using the EDGE scoring approach^10^. A dated phylogenetic tree comprising 923 species (84.7% of species diversity with at least one representative of each family and genus; Supplementary Table S1) was inferred from publicly available and newly-generated plastid and nuclear DNA sequences. The phylogenetic tree was calibrated using a set of fossils and molecular estimates (Supplementary Table S2) and missing species were randomly added to their corresponding genus to obtain a species-level tree of gymnosperms (see Materials and methods section). The majority of species (92.1%; 1,004 of the 1,090 species) have IUCN Red List assessments (version 2015.4; accessed 29 April 2016) and our sampling covers 89.3% (897 species) of the species currently evaluated. Of the 1004 assessed species, 401 (39.9%) are threatened (Vulnerable, VU, 156; Endangered, EN, 161; Critically Endangered, CR, 80; Extinct in the Wild, EW, 4), and 583 are non-threatened (Near Threatened, NT, 167; Least Concern, LC, 416), while 20 are currently listed as Data Deficient (DD). Eighty-six species have not yet been evaluated (NE). Risk of extinction was taken into account by converting IUCN Red List categories into probabilities of extinction using the original logarithmic transformation of Isaac and colleagues (hereafter “ISAAC”)^10^ and the IUCN50 transformation (hereafter “IUCN50”) suggested by Mooers and colleagues^30^.

**Figure 1:**
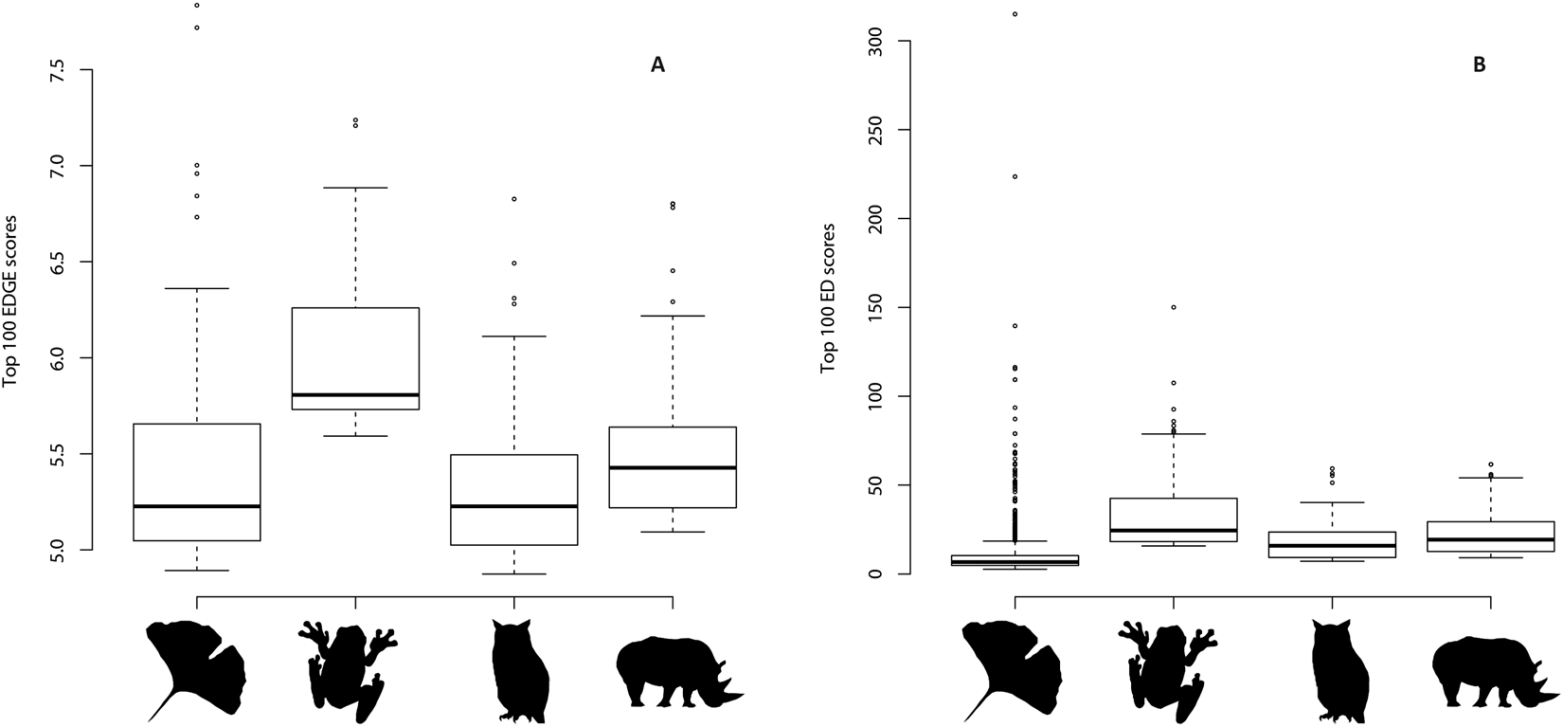
Comparative boxplots of EDGE scores (**A**) and ED values (**B**) for the top 100 EDGE-ranked species of (from left to right) gymnosperms, amphibians, birds, and mammals. EDGE scores for gymnosperms are those obtained using the ISAAC transformation and those for the three vertebrate groups were obtained from http://www.edgeofexistence.org/.

The top 100 EDGE scores obtained for gymnosperms (Supplementary Table S3) were compared to those published for mammals^10,11^, birds^12^, and amphibians^14^ (EDGE scores not available for sharks and relatives^15^ at the time). Although the ED scores of the top 100 EDGE gymnosperms are overall lower than those observed for the three animal groups, the number and distribution of outlier ED scores exceed those obtained for the animal groups (Fig. 1). These outlier species should be considered priority species for conservation. On the other hand, the EDGE values of the top 100 gymnosperms are more comparable to those recovered for the three animal groups, with medians hovering around the same value, except maybe for amphibians (Fig. 1). This indicates that the stability of species rankings obtained using the original EDGE approach^10^ remains to be evaluated and compared to rankings achieved under other extinction probability transformations of the IUCN Red List categories. It is possible that different transformations would better reflect the threats that species face and provide a ranking sufficiently stable to allow the prioritization of species for long-term conservation programs^30^.

The IUCN50 transformation^30^ attribute a lower extinction risk to non-threatened species and a higher extinction risk to threatened species compared to the ISAAC transformation^10^. Here, IUCN50 was favoured for gymnosperms because it better reflects the high level of threats faced by this group (ca. 40% of species are threatened based on the IUCN Red List) and, consequently, their need for urgent conservation actions. The larger difference between probabilities of extinctions of non-threatened (LC = 0.00005, NT = 0.004) and threatened (VU = 0.05, EN = 0.42, CR = 0.97) species under IUCN50 could diminish the contribution of ED values to the overall EDGE ranking. On the other hand, the ISAAC transformation is less sensitive to this issue (LC = 0.025, NT = 0.05; VU = 0.1, EN = 0.2, CR = 0.4) and might therefore favour species with high ED values compared to IUCN50, irrespective of the threat they face. Nevertheless, the top nine EDGE species are the same under both transformation scenarios, which underlines the uniqueness of these species and their conservation value (Table 1). The only difference within the top nine species is the position of the monotypic genera *Wollemia* (*W. nobilis*) and *Ginkgo* (*G. biloba*), with *G. biloba* found in first position under the ISAAC transformation, while *W. nobilis* occupies the first rank when the IUCN50 transformation is used (Table S3). Three of the top 20 EDGE species under ISAAC are not found among the top 20 species under IUCN50: *Sciadopitys verticillata* (NT; ISAAC, 10^th^; IUCN50, 385^th^), *Parasitaxus ustus* (VU; ISAAC, 12^th^; IUCN50, 139^th^), and *Taiwania cryptomerioides* (VU; ISAAC, 20^th^; IUCN50, 212^th^). These greatly fluctuating rankings demonstrate how ISAAC favours ED compare to IUCN50, which is particularly salient in the case of *S. verticillata* (see Supplementary Table S3). Among the top 100 EDGE species (under IUCN50), 84 (28 CR, 33 EN, 7 DD, 16 NE) overlap between the two approaches, which indicates some validity in the results obtained from the EDGE method (Supplementary Fig. S1; Supplementary Table S3).

**Table 1:** List of the top 20 EDGE species of Gymnosperms, with distribution, IUCN Red List assessments, evolutionary distinctiveness (ED) values (and associated rank), and evolutionary distinctive and global endangered (EDGE) scores (using IUCN50 transformation).

Rank	Taxon	IUCN	Distribution	Median ED	ED Rank	EDGE
1	*Wollemia nobilis* (Araucariaceae)	CR	Australia	139.59	3	4.91
2	*Ginkgo biloba* (Ginkgoaceae)	EN	China	315.00	1	4.89
3	*Araucaria angustifolia* (Araucariaceae)	CR	Brazil to Argentina	67.71	16	4.18
4	*Agathis australis* (Araucariaceae)	NA	New Zealand	64.76	18	4.14
5	*Acmopyle sahniana* (Podocarpaceae)	CR	Fiji	57.56	26	4.02
6	*Pherosphaera fitzgeraldii* (Podocarpaceae)	CR	Australia	51.44	35	3.91
7	*Glyptostrobus pensilis* (Cupressaceae)	CR	China to Laos	35.17	53	3.53
8	*Microcycas calocoma* (Zamiaceae)	CR	Cuba	30.82	66	3.40
9	*Araucaria araucana* (Araucariaceae)	EN	Chile to Argentina	67.71	17	3.35
10	*Cephalotaxus alpina* (Taxaceae)	NA	China	26.95	80	3.26
11	*Cunninghamia konishii* (Cupressaceae)	EN	Indo-China, China, Taiwan	52.12	32	3.09
12	*Metasequoia glyptostroboides* (Cupressaceae)	EN	China	51.26	37	3.07
13	*Torreya taxifolia* (Taxaceae)	CR	Georgia to Florida	21.63	126	3.04
14	*Agathis montana* (Araucariaceae)	CR	New Caledonia	21.09	131	3.02
15	*Sequoia sempervirens* (Cupressaceae)	EN	Oregon to California	47.81	42	3.00
15	*Sequoiadendron giganteum* (Cupressaceae)	EN	California	47.81	42	3.00
17	*Taxus floridana* (Taxaceae)	CR	Florida, Mexico	20.20	143	2.97
18	*Prumnopitys standleyi* (Podocarpaceae)	EN	Costa Rica	46.23	45	2.97
19	*Araucaria nemorosa* (Araucariaceae)	CR	New Caledonia	18.29	159	2.88
20	*Taxus florinii* (Taxaceae)	NA	China	18.15	161	2.87

Note that *Sequoia* and *Sequoiadendron* both occupy the 15^th^ rank as they are sister taxa and both EN, thus they have the same ED and EDGE scores.

**Figure 2:**
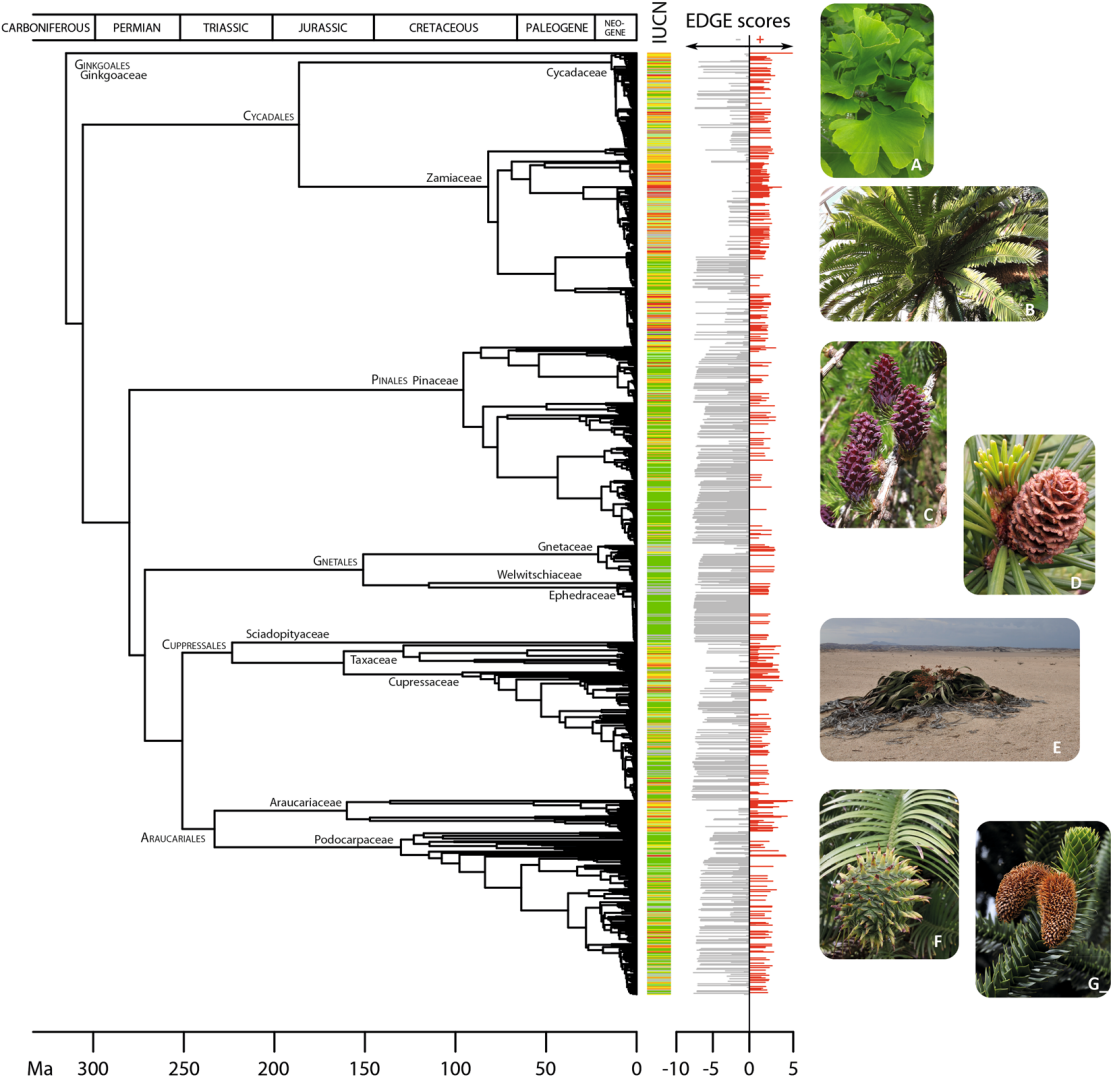
Gymnosperm dated tree (angiosperms, ferns, and fern allies have been pruned) inferred from plastid and nuclear DNA regions and comprising 923 species (ca. 85% of the group’s total species diversity) to which the 167 missing species were added randomly within their respective genera (see text for details). Orders and families are indicated. IUCN Red List assessments are colour-marked on the right of the tree (purple, Extinct in the Wild; red, Critically endangered; orange, Endangered; yellow, Vulnerable; light green, Near Threatened; dark green, Least Concern; grey, Data Deficient and Not Evaluated). EDGE scores are indicated. A selection of gymnosperms species, with their EDGE rank and threatened species ED rank (if applicable): (**A**) *Ginkgo biloba*, (Ginkgoaceae; EDGE 2^nd^, ED 1^st^); (**B**) *Encephalartos altensteinii* (Zamiaceae; EDGE 404^th^, ED 128^th^); (**C**) *Larix decidua* (Pinaceae; EDGE 745^th^; ED n/a); (**D**) *Sciadopitys verticillata* (Sciadopityaceae; EDGE 385^th^, ED n/a); (**E**) *Welwitschia mirabilis* (Welwitschiaceae; EDGE 675^th^; ED n/a); (**F**) *Wollemia nobilis* (Araucariaceae; EDGE 1^st^, ED 2^nd^); (**G**) *Araucaria araucana* (Araucariaceae; EDGE 9^th^, ED 9^th^).

Eighteen of the top 20 EDGE species belong to the conifer families Araucariaceae (monkey puzzles), Cupressaceae (cypresses, redwoods), Podocarpaceae (yellowwoods), and Taxaceae (yews). The gymnosperm with the highest ED value score is *G. biloba* with 315.0, while the second, the Australian endemic *W. nobilis*, has a score of 139.59 (about 2.25 times smaller; Table 1). Their EDGE scores on the other hand are more similar, with *W. nobilis* just slightly higher (4.89 for *G. biloba* vs 4.91 for *W. nobilis*). *Wollemia nobilis*, the Wollemi pine, was discovered in the Blue Mountains of Australia in 1994, in narrow sandstone ravines where a warm temperate rainforest climate prevails. It has been labelled as a living fossil by some because its pollen is almost identical to the extinct Turonian genus *Dilwynites*^31,32^. The second rank obtained for *Ginkgo biloba* is largely due to its isolated position as the only member of the order Ginkgoales and as sister to the remainder of gymnosperms in our analysis (Fig. 2). Although widely cultivated, only a few Chinese natural populations of this tree remain^33^. Even without considering its extinction risk, the unique evolutionary history of *Ginkgo*, and the fact that it is the sole living representative of a once highly diverse group of species^34^, make its conservation a top priority. Third is *Araucaria angustifolia*, another member of Araucariaceae, found in Brazil and Argentina where its range has decreased by 97% in the last century, granting it the Critically Endangered status. A third member of Araucariaceae on the EDGE list is found in fourth position, the kauri tree, *Agathis australis* from northern New Zealand; the conservation status of this species has not been formally assessed. Fifth is *Acmopyle sahniana*, one of two species in the genus, with fewer than 200 mature individuals remaining on the islands of Fiji^35^. The first member of gnetophytes on the list is the tropical Indonesian/Malaysian *Gnetum ridleyi* (27^th^), listed as data deficient due to the sparse information available for this species. The highest placed cycad on the list is *Microcycas calocoma* (8^th^), the sole species of this genus endemic to Cuba.

The EDGE ranks for cycads produced by another study^16^ were compared to those obtained here (Table S6). Except for a few outliers, the EDGE ranks obtained for each species by the two studies are generally equivalent (Fig. S3A) and the difference between the two rankings for most species falls within a relatively narrow distributional range (75% of species have a ranking difference of 50 or less; Fig. S3B). Many of the larger differences encountered between the two EDGE rankings are likely due to the different phylogenetic placement of certain species and the broader phylogenetic framework applied in our study (i.e. EDGE values for cycads calculated in the context of all gymnosperms and with 80% of the species represented by DNA sequence data, instead of separately and with 58% of species with DNA sequence data as in previous study^16^). Most notable perhaps is that among the 16 species for which the ranking diverged the most between the two studies (i.e. more than 150 rank difference; see highlighted species in Table S6 and Fig. S3A), 13 are not represented by sequence data, i.e. they have been added subsequently to the phylogenetic trees in both studies. These large differences in ranking could be explained by the element of randomness involved in the addition of missing species. The higher the number of species without genetic data that require to be incorporated following the tree inference step, the less reliable will be the overall ED/EDGE ranking for this particular group.

In terms of taxonomic coverage, ISAAC selected 16 species, including 11 species from 10 genera, not represented in the top 100 species obtained with IUCN50 (Supplementary Table S4). Although we favour IUCN50, ISAAC does identify species currently considered as VU or NT, which exhibit unique evolutionary distinctiveness. Five of the species on this list are ranked in the top 10 in terms of ED value, and all of them are in the top 100 ED values. The uniqueness of these species highlights the potential evolutionary loss involved if they become extinct and the important effect a change in their conservation status would have on the EDGE ranking. Included among them is the Japanese endemic *Sciadopitys verticillata*, the only extant representative of Sciadopityaceae, which exhibits the second highest ED value after *G. biloba*. Another notable species on this list is the only parasitic species of gymnosperm, *Parasitaxus ustus* (Podocarpaceae), which has the 6^th^ highest ED value and ranked 12^th^ based on the ISAAC transformation. This shrub is restricted to the island of New Caledonia, where it seems to have only one host, *Falcatifolium taxoides*^36^ (ranked 711^th^ in our EDGE list), another member of Podocarpaceae endemic to New Caledonia.

Given that EDGE scores are sensitive to the probability of extinction attributed to each IUCN category (see above), we also examined the ED ranking of threatened species, i.e. species that have been assigned the IUCN categories CR, EN, or VU (we also include in this list the species that are either NE and DD, as we considered them as CR for our analyses). Only 10 of the top 20 EDGE species are also found in the top 20 ED threatened species. The other 10 species found in the top 20 ED threatened species list have all been assessed as VU (Table 2); these species are ranked between the 139^th^ and 301^st^ positions in the EDGE list. This further demonstrates the sensibility of EDGE scores to the probability of extinction that is assigned to IUCN categories. *Wollemia nobilis, Ginkgo biloba* and *Parasitaxus ustus* (respectively 1^st^, 2^nd^ and 139^th^ on the EDGE list) occupy the first three positions on the top 20 ED list of threatened species (Table 2). They are followed by two species that are found much further down the EDGE list, *Taiwania cryptomerioides* (Cupressaceae) and *Cathaya argyrophylla* (Pinaceae), respectively 212^th^ and 239^th^ on the EDGE list (Table 2). Both are species belonging to monotypic genera found in South East Asia. The first one, *T. cryptomerioides*, is one of the largest tree species in Asia, which was heavily exploited in the past leading to an estimated reduction of more than half of its original distribution range. The second, *C. argyrophylla*, is endemic to China and had once a much wider distribution, according to fossil records, but its natural populations are now reduced to a total of less than a thousand mature individuals.

**Table 2:** List of the top 20 ED threatened species of Gymnosperms, with distribution, IUCN Red List assessments, evolutionary distinctiveness (Median ED) values and associated overall rank, and evolutionary distinctive and global endangered (EDGE) scores (using IUCN50 transformation) and associated rank.

Rank	Taxon	IUCN	Distribution	Median ED	Overall ED rank	EDGE	EDGE rank
1	*Ginkgo biloba* (Ginkgoaceae)	EN	China	315.00	1	4.89	2
2	*Wollemia nobilis* (Araucariaceae)	CR	Australia	139.59	3	4.91	1
3	*Parasitaxus ustus* (Podocarpaceae)	VU	New Caledonia	109.34	6	1.70	139
4	*Taiwania cryptomerioides* (Cupressaceae)	VU	China, Eastern Asia, Indo-China	87.20	9	1.47	212
5	*Cathaya argyrophylla* (Pinaceae)	VU	China	72.43	12	1.29	239
6	*Pseudolarix amabilis* (Pinaceae)	VU	China	68.65	13	1.23	249
7	*Pseudotaxus chienii* (Taxaceae)	VU	China	68.23	15	1.23	250
8	*Araucaria angustifolia* (Araucariaceae)	CR	Brazil to Argentina	67.71	16	4.18	3
8	*Araucaria araucana* (Araucariaceae)	EN	Chile to Argentina	67.71	17	3.35	9
10	*Agathis australis* (Araucariaceae)	NE	New Zealand	64.76	18	4.14	4
11	*Prumnopitys ladei* (Podocarpaceae)	VU	Australia	58.72	23	1.08	273
12	*Acmopyle sahniana* (Podocarpaceae)	CR	Fiji	57.56	26	4.02	5
13	*Cunninghamia konishii* (Cupressaceae)	EN	Indo-China, China, Taiwan	52.12	32	3.09	11
14	*Stangeria eriopus* (Zamiaceae)	VU	South Africa	52.06	34	0.96	292
15	*Pherosphaera fitzgeraldii* (Podocarpaceae)	CR	Australia	51.44	35	3.91	6
16	*Metasequoia glyptostroboides* (Cupressaceae)	EN	China	51.26	37	3.07	12
17	*Araucaria heterophylla* (Araucariaceae)	VU	Norfolk Islands	49.50	40	0.91	298
18	*Prumnopitys andina* (Podocarpaceae)	VU	Chile to Argentina	49.02	41	0.90	301
19	*Sequoia sempervirens* (Cupressaceae)	EN	Oregon to California	47.81	42	3.00	15
19	*Sequoiadendron giganteum* (Cupressaceae)	EN	California	47.81	43	3.00	16

Note that *Araucari*a *angustifolia* and *A. araucana* both occupy the 8^th^ rank and *Sequoia* and *Sequoiadendron* the 19^th^ rank, as these taxon pairs are sister taxa and thus have the same ED.

The species that are either Data Deficient (DD) or that have not been evaluated (NE) were included in our principal EDGE analysis by assigning them a preliminary Critically Endangered (CR) status. Studies have shown that the majority of mammals considered as DD are more likely to be threatened than the species that have been already assessed^37,38^. In order to avoid the possibility of overlooking priority species, we explored how EDGE rankings would be impacted by assigning DD/NE species with the highest threat category CR, the worst-case scenario. Three of these species occur in the top 20 EDGE species, if their status is confirmed as CR (Table 1). This result clearly highlights the urgency of evaluating extinction risk for species that have not yet been assessed to ensure that conservation resources are adequately allocated. Likewise, it showcases the potential of phylogenetic data to identify species with unique evolutionary histories, but with little or no information regarding the threats they face, especially in the case of large groups of organisms such as angiosperms, fungi and insects, for which conservation assessments remain relatively scarce.

**Figure 3:**
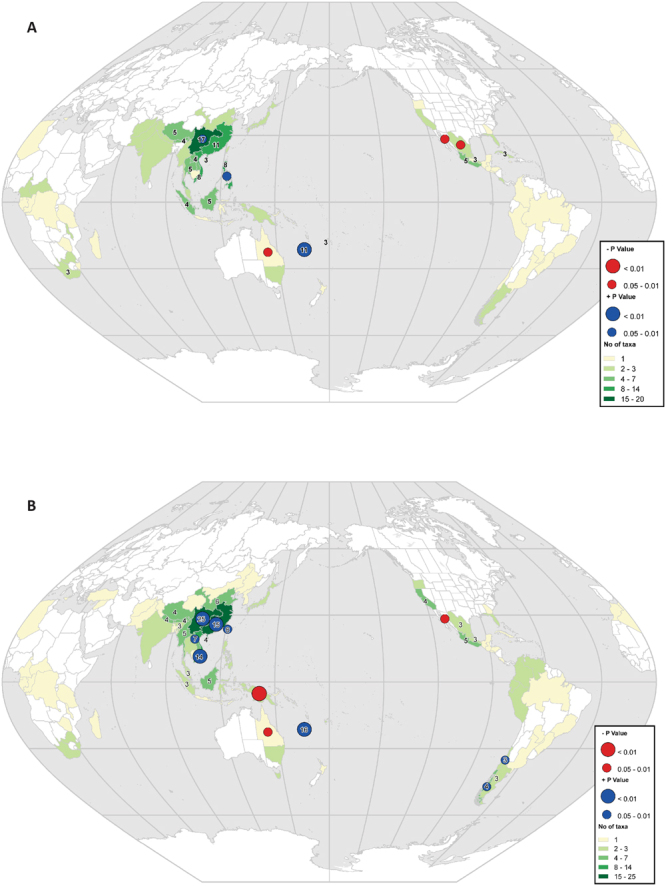
Distribution of (**A**) the top 100 EDGE species (based on the IUCN50 transformation; see text) and (**B**) top 100 ED threatened species, matched to the Taxonomic Databases Working Group (TDWG) geographical scheme level 3 geography^68^. Red circles identify areas with fewer EDGE/ED species and blue circles identify areas with more EDGE/ED species than expected; species distribution data were obtained from the World Checklist of Selected Plant Families. Data was displayed and processed in ArcGIS 10.1^69^, using the Winkel I projection orientated around the date line (180 degrees).

The inclusion of spatial information in our analyses highlighted three regions with a high number (>10%) of top 100 EDGE species: South-Central China (17 spp.), Southeast China (11 spp.) and New Caledonia (11 spp.; Fig. 3A). Only New Caledonia has a high number of species with significantly more species in the top 100 EDGE species than expected by chance (Exact Binomial Test; Fig. 3A). This may be explained by the accumulation on this archipelago of species of Podocarpaceae and Araucariaceae, which are older lineages than those found in other regions with similar species numbers (Figs 2 and 3). A similar analysis performed on the 100-threatened species with the highest ED values showed that the same three regions (South-Central China, 25 spp.; Southeast China, 15 spp.; New Caledonia; 16 spp.), joined by a fourth region, Vietnam (14 spp.), have each more than 10% of the top ED threatened species (Fig. 3B). South-Central China, Southeast China, New Caledonia and Vietnam have high species numbers and significantly more species in the top 100 ED threatened species than expected by chance (Exact Binomial Test; Fig. 3B). Their identification by both metrics (EDGE and ED of threatened species) highlights the importance of these regions for the global conservation of gymnosperms. These areas have suffered high rates of deforestation and since gymnosperms are generally important components of the ecosystem, conserving species with high EDGE scores and/or threatened species with high ED values would also contribute to the survival of other species and maintain the functioning of ecosystems (e.g. 70% of threatened Chinese vertebrates are affected by habitat destruction^39^). Notably, two of the five bird species with top EDGE scores^12^, the owlet-nightjar and the kagu, are endemic to New Caledonia, further emphasizing the importance of this hotspot for conservation. The distribution of the top 100 EDGE species contrasts with those of total and threatened species richness, with regions such as Mexico (Northeast and Northwest) and Queensland (Australia) having a high number of species, but with few of these among the top 100 EDGE species (Fig. 3A). Likewise, three regions with relatively large numbers of species (New Guinea, Northwest Mexico, Queensland), have no or only few of the top 100 ED threatened species (Fig. 3B). This can be explained by a large proportion of the species diversity in such regions resulting from the accumulation of recent lineages that contribute less to evolutionary distinctiveness. One other region has a low number of species in the top 100 EDGE species, but nevertheless have significantly more species than expected by chance, Philippines. The same situation is also observed for four regions with low number of species that have significantly more species in the top 100 ED threatened species than expected by chance (South Chile, Central Chile, Laos, Taiwan).

We opted here for the original EDGE approach to facilitate the comparison with other groups. The EDGE approach has been criticized because it considers each species independently and thus ignores the actual risk of extinction associated with internal branches. The internal branches are assigned a risk of extinction dependent of the taxon assessed only, irrespective of the threats potentially faced by the other taxa subtended by this internal branch. In other words, the shared responsibility for the survival of a given internal branch is not accounted for under the original EDGE method^40^. Nonetheless, under simple evolutionary models and random extinction across phylogenetic trees, recent simulations have shown that the loss of ED is correlated to the loss of PD^41,42^. Other approaches using the concept of “expected PD”^40,43–45^ and building on EDGE have been developed that consider the risk of extinctions faced by closely-related taxa, although in some cases their effects are not revealed in the top species of a given list (e.g.^45–48^). It is also important to note that even though EDGE and threatened ED have a large overlap in the regions they highlight as important for gymnosperms (see above; Fig. 3), the number of EDGE species found in a given area does not necessarily relate to the amount of threatened PD nor the expected PD gained if these species were to be secured^49^.

The protection of species with the highest EDGE scores would ensure the preservation of key lineages representing unique evolutionary features within gymnosperms. The list provided here is dynamic. The availability of new DNA sequence data, new or revised assessments and, eventually, the more general implementation of methods based on expected PD, is likely to identify additional priorities for conservation. Importantly, this method provides a valuable baseline against which to measure the impact of conservation programs on gymnosperms. Urgent actions are paramount in the face of increasing anthropogenic pressures on both species and ecosystems. The integration of evolutionary history in biodiversity science is thus more vital than ever to achieve effective conservation^50–53^ and approaches such as EDGE provide a mean to prioritise, accelerate and optimise conservation actions by accounting for the overall evolution of a species and the threats it faces.

## Materials and Methods

A list of all gymnosperm species was obtained from the Royal Botanic Gardens, Kew online resource “World Checklist of Selected Plant Families”^17^. Available DNA sequence data for gymnosperms for the plastid regions *rbcL*, *matK*, *rpoC*, *rps4*, and *trnL*, as well as the nuclear marker *PHYP*, were obtained from GenBank and downloaded using the data-mining tool SUMAC^54^ (data accessed on 3^rd^ March 2016). Forty-one taxa of angiosperms and fifteen ferns and their allies were also included in our analyses as outgroup taxa. Regions were selected based on the level of coverage they achieved either across gymnosperms as a whole or with a focus on particular lineages. Details of species sampled for each region (including GenBank accession numbers) are listed in Supplementary Table S1.

To increase taxonomic coverage, we obtained sequence data for the plastid *rbcL* exon (ribulose-1,5-bisphosphate carboxylase/oxygenase large subunit) for 129 species, of which 35 were for species otherwise not represented in the data set. DNA was isolated using a modified version of the 2× CTAB method^55^ and subsequently purified on a caesium chloride/ethidium bromide gradient (1.55 g/ml density) to yield material suitable for long-term storage in the DNA & Tissue Collections at the Royal Botanic Gardens, Kew (http://apps.kew.org/dnabank/homepage.html). PCR amplifications were performed using primer combinations from Olmstead and colleagues^56^. PCR reactions were made with the ReddyMix PCR Master Mix from ABgene (2.5 mM MgCl2; Epsom, Surrey, UK) with the addition of 1 μl of bovine serum albumin 0.4% and 50 ng of each primer, in a final volume of 25 μl. The amplification cycle started with 2 min initial denaturation at 94 °C, followed by 32 cycles of 1 min denaturation at 94 °C, 1 min annealing at 48 °C, 1.5 min extension at 72 °C, and a final extension of 3 min at 72 °C. After purification with the Nucleospin Extract II kit (Machery-Nagel, Duren, Germany), cycle sequencing reactions were performed in 10 μl reactions using 1 μl of BigDye® Terminator cycle sequencing chemistry (v3.1; ABI; Warrington, Cheshire, UK) and run on ABI 3730 automated sequencer. Geneious^57^ (version 7.1.2) was used to assemble complementary strands and verify base-calling.

Sequences of each region were compiled in Geneious^57^ (version 7.1.2) and aligned using the MUSCLE^58^ algorithm. All partitions were concatenated using an R script (S. Buerki, pers. comm.) and all subsequent analyses were performed on the resulting supermatrix. A phylogenetic tree was reconstructed using the maximum likelihood (ML) criterion as implemented in the software RAxML (v. 8.2.8^59^) on the CIPRES portal (www.phylo.org) with 1,000 rapid bootstrap replicates followed by the search of the best ML tree. The GTRCAT model was used and all the other parameters were set as default settings. All fifteen ferns and allies were designated as outgroup taxa (e.g.^20,22^).

Several attempts to obtain an ultrametric tree using the Bayesian approach implemented in the package BEAST^60^ were unsuccessful. Constraining the topology to the ML tree obtained from the software RAxML, thus allowing only the optimisation of branch lengths alone, was also unsatisfactory. In all cases, the analyses failed to converge on a single solution and the majority of effective sample size values were consistently below the threshold of 200. We thus opted to transform the ML phylogenetic tree of gymnosperms into an ultrametric tree using the programme *treePL*^61^, which implements the penalized likelihood method^62^. The default cross validation procedure was performed and identified 0.1 as the most appropriate smoothing value. A set of 15 calibration points based on fossils used by previous studies and molecular estimates from a recent study of cycads were applied (see Supplementary Table S2). Outgroup taxa were pruned from the tree prior to the calculation of ED scores.

Despite having a reasonably good species coverage in our phylogenetic analysis (i.e. ca 85%), incomplete sampling could potentially biased EDGE rankings, thus we used the following approach to add to our ultrametric tree the 167 species for which no suitable sequence data was available for the markers used here. We used the function *add.species.to.genus* from the R^63^ package *phytools*^64^ and the option “random”, which add randomly the missing species to their respective genera, while retaining the ultrametricity of the tree. We performed this step 100 times to assess how the random position assigned to each species within its genus affects the ED and EDGE values, and the resulting EDGE ranks.

ED scores for all species of gymnosperms were obtained using the 100 ultrametric trees and were inferred using the function *evol.distinct* from the R^63^ package *picante*^65^. The median value of all 100 resulting ED values for each species was compiled and used to produce the EDGE scores. Probability of extinction assessments were obtained from the IUCN Red List (www.iucnredlist.org, version 2015.4; accessed on 29^th^ April 2016). These assessments were converted into probabilities of species extinction using two probability of extinction transformations, the original logarithmic transformation of Isaac and colleagues^10^, and the IUCN50 probability transformation proposed by Mooers and colleagues^30^. EDGE scores were subsequently calculated using the median ED value by implementing the EDGE equations in an R^63^ script. Species that were Data Deficient (DD) or Not Evaluated (NE) were scored as Critically Endangered. Threatened species (i.e. those assigned CR, EN, VU, as well as DD and NE) were ranked by decreasing ED scores to provide a classification conservation priority species less dependent on the transformation of probability of extinction.

The gymnosperm species with the top 100 EDGE values obtained with the ISAAC transformation together with their ED scores were compared to those of amphibians, mammals and bird (obtained from www.edgeofexistence.org) using boxplots produced in R^63^. We compared the effect of probability of extinction transformations (IUCN50 vs. ISAAC) on the overall EDGE species ranking by plotting the difference in species rankings using the IUCN50 transformation as reference; negative values indicate that the IUCN50 transformation prioritize a given species over the ISAAC transformation, whereas positive values denote the opposite. Differences in EDGE species rankings were plotted using R^63^ and each species was coloured according to its IUCN Red List category. To assess the effect of ED on EDGE species ranking, boxplots of ED values for the species prioritized by each transformation were also produced in R^63^. A difference of ranking between plus or minus 10 was considered equivalent for the boxplot (following^30^). A figure displaying the gymnosperm dated tree together with EDGE values (inferred using the IUCN50 transformation) and IUCN Red List assessments was produced in R^63,64^. The GSA geological time scale was used to set boundaries between geological periods^66,67^.

To map gymnosperm diversity, data from the World Checklist of Selected Plant families^17^ (accessed 30 August 2016) were matched to the Taxonomic Databases Working Group (TDWG) geographical scheme level 3 geography^68^. Data was displayed and processed in ArcGIS 10.1^69^, using the Winkel I projection orientated around the date line (180 degrees) and to give an interpretable and reproducible map, colours were derived from Color Brewer^70^. To evaluate if the mapped ranking follows what is expected by chance, we used Exact Binomial Test performed in R^63^ against the top 100 EDGE species using the IUCN50 transformation, assuming that the number of top 100 species in each TDWG level 3 region is expected to be proportional to the observed total number (species richness). We repeated the same analysis with the top 100 ED threatened species. The overall result (all TDWG regions) was not significant, but was highly significant for some of the individual TDWG regions, with either more or fewer species than expected by chance (see Fig. 3A).

### Data availability

All newly-produced DNA sequences were deposited in GenBank (accession numbers MH069511-MH069638). The combined matrix and phylogenetic trees are available at https://treebase.org/treebase-web/home.html (Submission 21792).

## Electronic supplementary material

Supplementary figures

Supplementary tables
