# Supplementary material for: Gymnosperms on the EDGE
Source: Sci Rep. 2018 Apr 16;8:6053. doi: 10.1038/s41598-018-24365-4 (PMC5902588; doi:10.1038/s41598-018-24365-4)
Supplement: Supplementary file 1 — Supplementary figures [file 41598_2018_24365_MOESM1_ESM.docx]

**Supplementary Figures S1-S3**

**Gymnosperms on the EDGE**

Félix Forest, Justin Moat, Elisabeth Baloch, Neil A. Brummitt, Steve Bachman, Steffi Ickert-Bond, Peter M. Hollingsworth, Aaron Liston, Damon P. Little, Sarah Mathews, Hardeep Rai, Catarina Rydin, Dennis W. Stevenson, Philip Thomas, Sven Buerki.

**Supplementary** **Fig. S1.** The effect of probability of extinction transformations (IUCN50^1^ vs. ISAAC^2^) on the overall EDGE species ranking. A) The difference in species rankings using the IUCN50 transformation as reference; negative values indicate that the IUCN50 transformation prioritize a given species over the ISAAC transformation, whereas positive values denote the opposite; each species was coloured according to its IUCN Red List category. B) Boxplots of ED values for the species prioritized by each transformation below and above the grey line (a difference of ranking between plus or minus 10) in Supplementary Fig. S1A. The IUCN50 approach mostly resulted in a species ranking within each IUCN category following their ED values (A). On the other hand, the analysis conducted with ISAAC allowed less threatened species exhibiting significantly higher ED values to rank higher than in the IUCN50 ranking (A; see also Supplementary Table S3). This difference between the two approaches is further confirmed by the comparison of ED scores and IUCN categories, where species prioritized using ISAAC have significantly higher ED scores than those prioritized with IUCN50 (B). Within the top 100 EDGE species, IUCN50 only included CR (36 spp.), EN (34 spp.) and DD/NE (30 spp.; treated as CR) species, whereas ISAAC included species that are VU (13 spp.) and NT (3 spp.) in addition to CR (28 spp.). EN (33 spp.) and DD/NE (23 spp.). Overall, the 84 species shared between the two correspond to the CR, EN and DD/NE species highlighted by ISAAC.


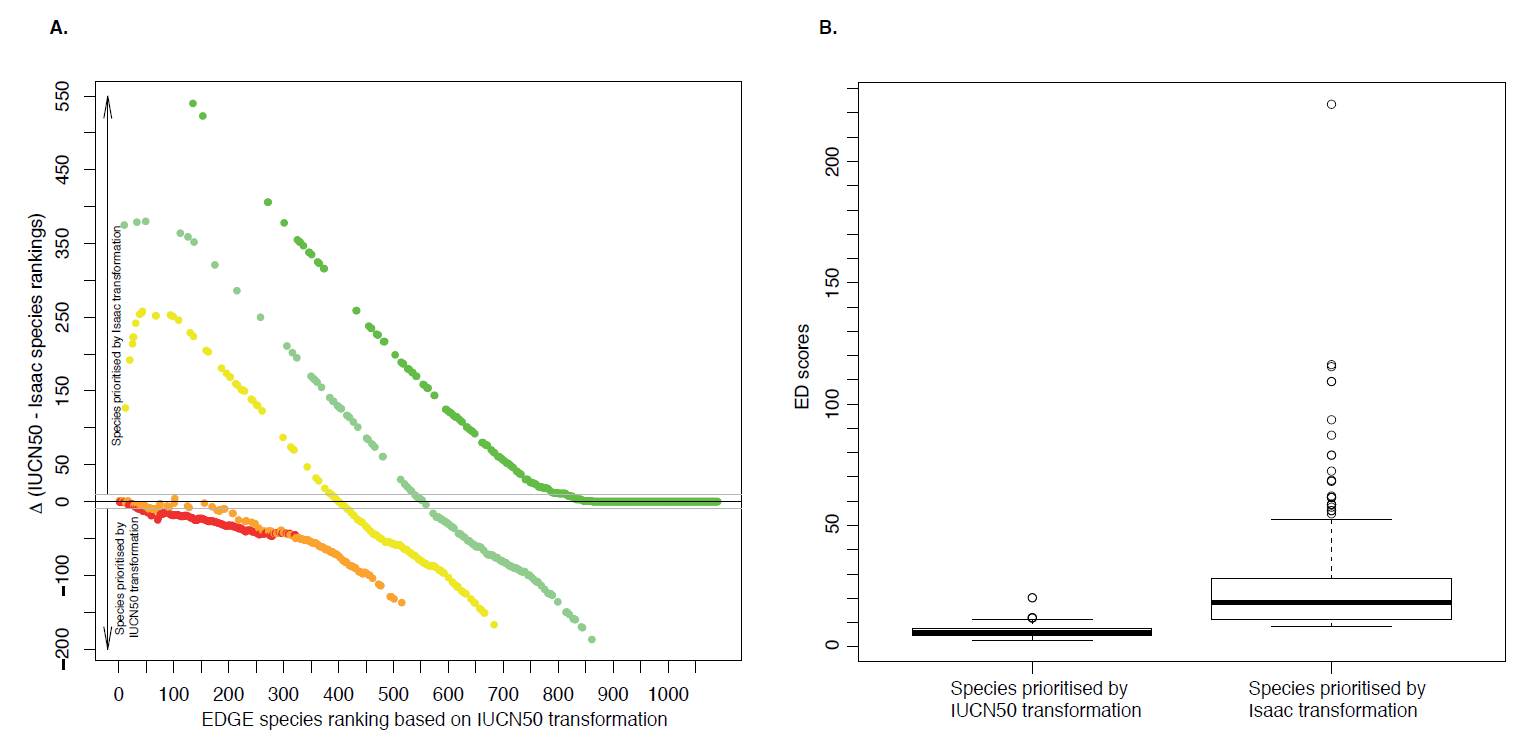


**Supplementary** **Fig. S2.** Distribution of gymnosperms based on the Taxonomic Databases Working Group (TDWG)^3^ geographical scheme level 3 of A) the richness for all species and; B) richness of threatened species. Numbers are species diversity within areas (regions with 0 to 15 species not labelled); species distribution data were obtained from the World Checklist of Selected Plant Families^4^. Data was displayed and processed in ARCGIS 10.1^5^, using the Winkel I projection^6^ orientated around the date line (180 degrees; see main text for details).

A)


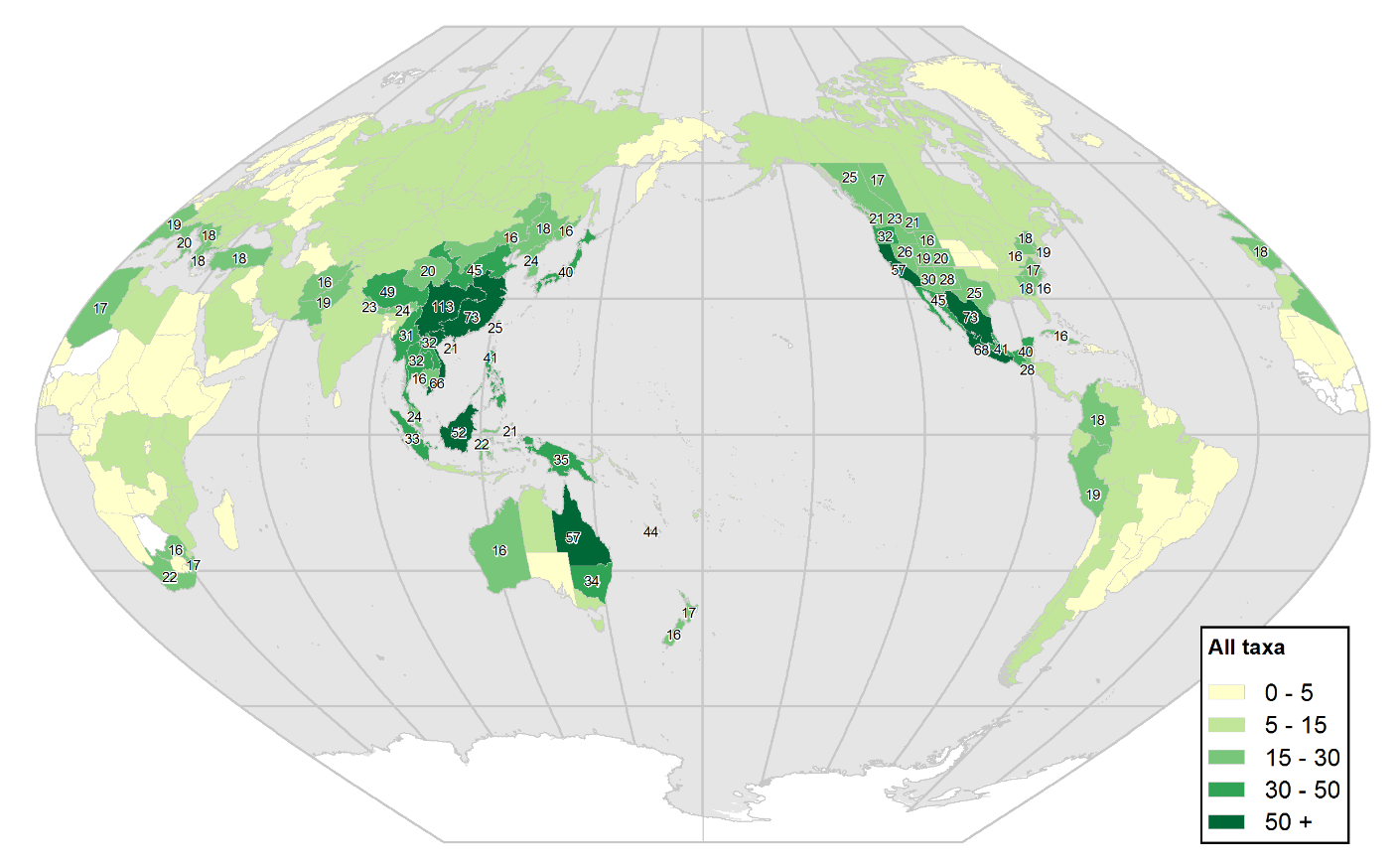


B)


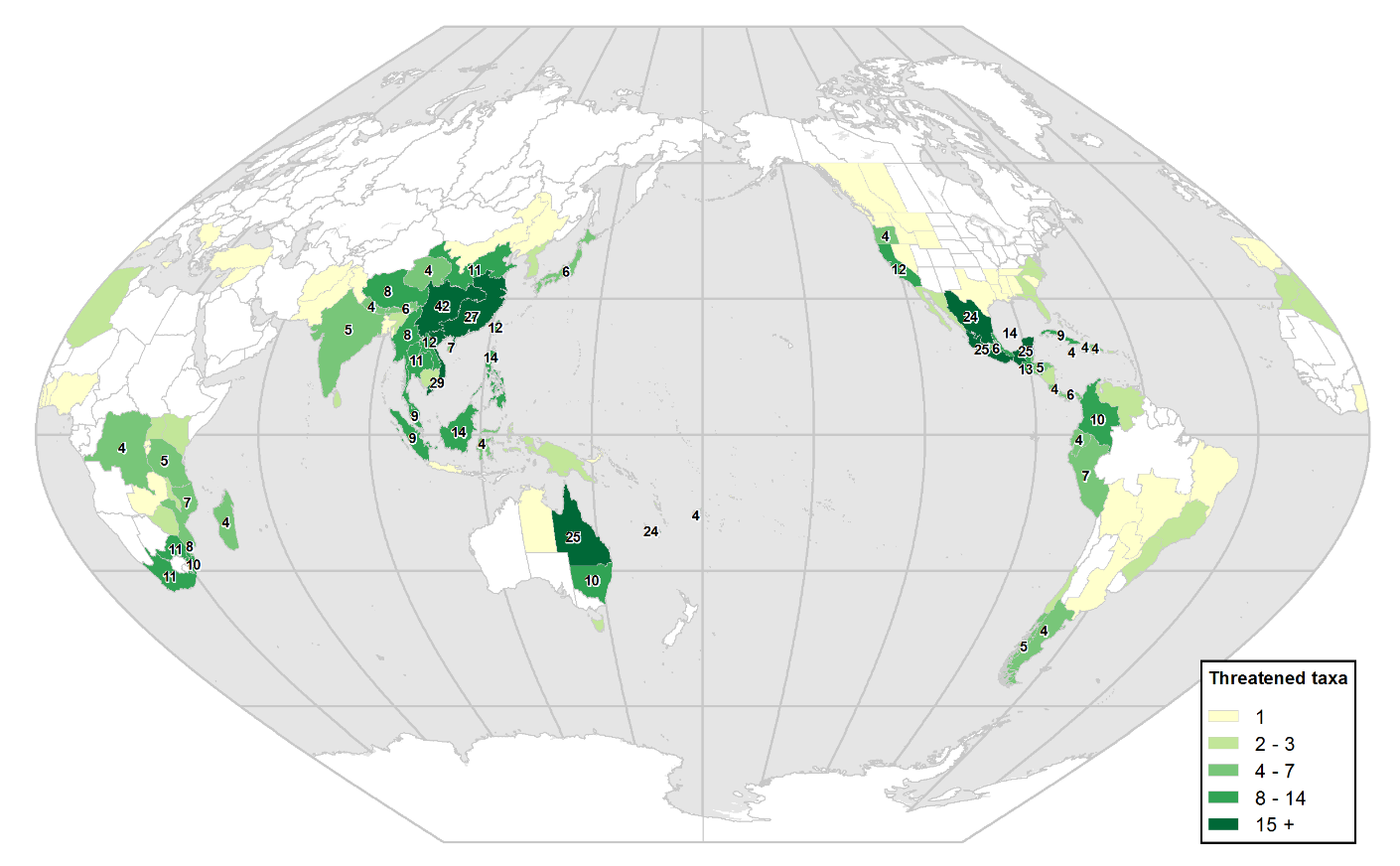


**Supplementary** **Fig. S3.** Comparison of EDGE ranks obtained by the present study and the study of Youssoufou et al^7^ focussing on cycads only. A) Ranks obtained by Yessoufou et al plotted against the ranks obtained by the present study. B) Histogram depicting the distribution of differences between the two rankings.


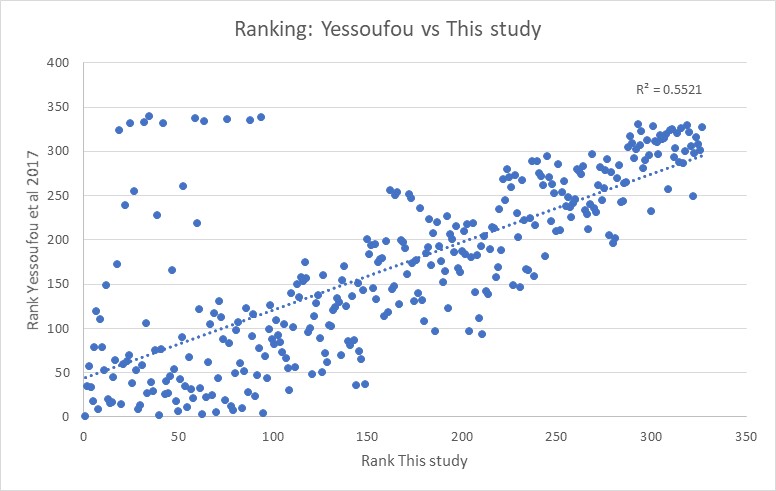
A)


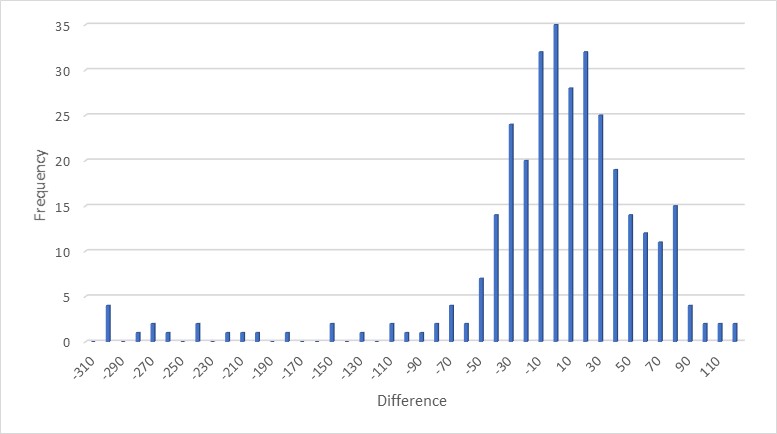
B)

**References**

1 Mooers, A. O., Faith, D. P. & Maddison, W. P. Converting endangered species categories to probabilities of extinction for phylogenetic conservation prioritization. *Plos ONE* **3**, doi:10.1371/journal.pone.0003700 (2008).

2 Isaac, N. J. B., Turvey, S. T., Collen, B., Waterman, C. & Baillie, J. E. M. Mammals on the EDGE: Conservation priorities based on threat and phylogeny. *Plos One* **2**, doi:10.1371/journal.pone.0000296 (2007).

3 Brummitt, R. K. *World Geographical Scheme for Recording Plant Distributions*. 2 edn, (Hunt Institute for Botanical Documentation, Carnegie-Mellon University, Pittsburgh, Penna. (for the International Working Group on Taxonomic Databases for Plant Sciences), 2001).

4 RBG Kew. *World Checklist of Selected Plant Families*, <http://apps.kew.org/wcsp/> (2016).

5 ArcGIS Desktop v. Release 10 (Environmental Systems Research Institute, Redlands, CA, 2012).

6 Brewer, C. A. <http://www.ColorBrewer.org> (2016).

7 Yessoufou, K., Daru, B. H., Tafirei, R., Elansary, H. O. & Rampedi, I. Integrating biogeography, threat and evolutionary data to explore extinction crisis in the taxonomic group of cycads. *Ecology and Evolution* **7**, 2735-2746, doi:10.1002/ece3.2660 (2017).
